# A comparison of body image components, including attentional bias and body appraisal, in adolescents with anorexia nervosa or depression, and non-clinical controls

**DOI:** 10.1186/s13034-026-01160-y

**Published:** 2026-08-24

**Authors:** Inken Kirschbaum-Lesch, Anne Radix, Laura Mokros, Anca Sfärlea, Belinda Platt, Gerd Schulte-Körne, Eni S. Becker, Martin Holtmann, Tanja Legenbauer

**Affiliations:** 1 LWL-University Hospital for Child and Adolescent Psychiatry and Psychotherapy, Ruhr-University Bochum, Heithofer Allee 64, Hamm, 59071 Germany; 2 Department of Child and Adolescent Psychiatry, Psychosomatics and Psychotherapy, University Hospital, LMU Munich, Munich, Germany; 3 Behavioural Science Institute, Radboud University Nijmegen, Nijmegen, Netherlands

**Keywords:** Body image disturbance, Adolescents, Depression, Attentional bias, Body dissatisfaction, Body appraisal, Shape concerns

## Abstract

**Background:**

Body image disturbance (BID) is increasingly recognized as a transdiagnostic factor. Beyond its established role in anorexia nervosa (AN), specific components of BID are also associated with the development and maintenance of psychopathology in adolescent Major Depressive Disorder (MDD). However, measurements of different body image components (perceptual, affective, cognitive, and behavioral)—especially objective behavioral measures—remain scarce; the assessment of attentional biases (AB) represents a promising tool to fill this gap.

**Methods:**

In the present study, two clinical groups of adolescent inpatients (MDD: *n* = 28; AN: *n* = 34) and non-clinical controls (NCC: *n* = 39; aged 13–19 years) completed a body-related Stroop task and a free-viewing task including a body appraisal. Participants also completed questionnaires assessing shape concerns, social appearance anxiety, body checking, avoidance, and reassurance.

**Results:**

The AN and the NCC groups revealed a significant body-related AB, whereas the MDD group did not. Regarding body appraisal, the clinical groups rated their bodies more negatively than the NCC group. On shape concerns, anxiety, and body avoidance/checking, the AN group showed elevated scores compared to the MDD group. Notably, the NCC only differed significantly from the AN group regarding shape concerns.

**Conclusions:**

The three groups exhibited distinct BID profiles, identifying pathological body appraisal as a transdiagnostic feature that suggests body dissatisfaction interventions could also mitigate adolescent depressive symptoms. Furthermore, the lack of behavioral differences between healthy adolescents and the AN group underscores the need for early prevention targeting checking and avoidance to prevent the progression into clinical eating disorders.

## Background

Body image is highly relevant for 84.1% of students [1]. A systematic review found that many adolescent girls were dissatisfied with their body weight, with the prevalence of body weight dissatisfaction ranging from 19.2% to 83.8% [2]. Longitudinal data shows that body image deteriorates between ages 10 and 16—particularly in girls (10–14)—before improving again towards age 24 [3]. This vulnerability in adolescence (10–19 years) stems from a convergence of pubertal changes, individuation from family, and increased peer-group orientation [2]. Furthermore, rising social media consumption intensifies exposure to idealized body standards, collectively driving the high prevalence of body dissatisfaction in this age group [2].

Slade, [4] defined body image as the mental representation of one’s size and shape, alongside the feelings associated with these characteristics. Accordingly, body image disturbances (BIDs) encompass perceptual, affective, cognitive, and behavioral components [5]. Body dissatisfaction, a core element of BIDs arises from a perceived discrepancy between one’s actual and ideal body, leading to negative thoughts and emotions [6]. Consequently, effective assessment of BIDs must incorporate multimodal measures covering affective, cognitive, and behavioral components [7].

BIDs have been extensively researched within the field of eating disorders (EDs). They represent a hallmark feature of Anorexia Nervosa [8] and are a well-known prognostic and risk factor in AN [7]. The most extensively investigated component of BID in AN is the perceptual component [7]. Research consistently reveals an overestimation of body dimensions, including weight, size, and shape, as well as other body-related metrics in AN (e.g., [9, 10]). Furthermore, physiological studies have identified altered visual processing patterns, characterized by more extensive gaze fixations and a modified visual scanning behavior that focuses predominantly on salient body regions [11, 12]. The emotional component of BIDs is characterized by an emotional attentional Bias (AB) during exposure to body-related imagery, often accompanied by altered interoceptive awareness [13].

With regard to the cognitive component, research has identified attentional, interpretation, and memory biases, as well as impaired executive functions (e.g., cognitive interference) when patients with AN are confronted with body-related stimuli [14, 15]. Especially attentional ABs – presenting as hypervigilance toward disorder-relevant (body) stimuli, which are preferentially processed due to their consistency with maladaptive schemata [16] - have been extensively studied in EDs. These biases are characterized by a delayed disengagement from body-related stimuli [7]. On the behavioral component, patients frequently engage in body checking (e.g., repeated mirror use, frequent weighing) or body avoidance (e.g., wearing oversized clothing; behavioral component; 17).

Recent studies have questioned the specificity of BIDs to AN. Lukas et al. [18] demonstrated that memory and interpretation biases for ED-related stimuli did not differ significantly between adolescents with AN and a clinical control group (mainly patients with Major Depressive Disorder; MDD). This lack of specificity was mirrored in a visual dot-probe task, where no significant differences emerged between patients with AN and patients with MDD regarding their response to underweight and overweight body images [19]. Instead, all groups—including non-clinical controls (NCCs)—showed body-related AB in this adolescent sample. Using an eye-tracking task in the same sample, Sfärlea et al. [20] found similar patterns when comparing attention to pictures of bodies versus faces. Consequently, these cognitive biases may represent transdiagnostic features rather than markers unique to EDs.

Clinical research increasingly highlights a robust association between BIDs and MDD [21, 22]. Longitudinal evidence identifies body-related factors as critical predictors for the development of adolescent MDD. Specifically, body dissatisfaction at age 14 significantly predicts the onset of depressive episodes by age 18 [23]. Furthermore, weight-reduction activities, the drive for muscularity, and the perception of being overweight serve as reliable predictive markers for MDD [24, 25]. This relationship is notably moderated by self-worth: greater weight-loss efforts are associated with more pronounced depressive symptomatology, particularly when self-esteem is low [25]. In contrast to AN, where body image is diagnostically central, BID in MDD may reflect a broader negative self-schema. In this context, body-related concerns might be domain-specific manifestations of the cognitive triad [26]—a hallmark feature of depression characterized by a negative view of oneself, the world, and the future.

These findings align with evidence stating that body-related factors, such as BID, play a crucial role not only in EDs but in adolescent MDD as well [27]. This raises the question of which body image components are specific to AN or MDD, and which represent normative adolescent development. Identifying the specific components that serve as risk factors for MDD or AN is crucial for understanding the mechanisms underlying the onset and maintenance of these disorders. Ultimately, such insights are essential for developing targeted preventive strategies and therapeutic interventions.

While previous research involving patients with MDD has primarily focused on isolated components of BID, the present study addresses this gap by comparing adolescent inpatients with MDD or AN to a NCC group on different components of BID. To complement the studies by Radix et al. [19] and Sfärlea et al. [20], which investigated ABs using pictorial body stimuli in and overlapping sample, the present study aims to explore ABs toward body-related words using the Stroop Task. The Stroop task [28] is an established method to measure AB in MDD [29] and EDs [30, 31] by comparing reaction times (RTs) between congruent and incongruent color-naming conditions. To assess disorder-specific AB, an “emotional Stroop task” using negatively valenced and body-related words is applied. Using words as stimuli allows for examining the generalizability of previous findings, from purely perceptual reactions to semantic processing. Furthermore, if AB occurs in response to words, it would suggest that these biases are more deeply rooted in abstract knowledge structures and the self-concept, rather than being merely a reaction to visual stimuli.

In addition, a body appraisal task following confrontation with one’s own body, and the shape concerns subscale of an eating disorder questionnaire (cognition) are used to assess self-reported body dissatisfaction. The body-related AB Stroop test measures implicit and automatic cognitive changes, while the body appraisal task and the shape concern subscale measure explicit and conscious body dissatisfaction. Additionally, the participants completed questionnaires to asses social appearance anxiety (affect), body checking, avoidance, and reassurance (behavior). To test the validity of the measures, linear correlations were conducted. If the BI components are indeed relevant, they should show a statistical association with eating disorder and depressive symptom severity.

## Materials and methods

This observational study aims to compare cognitive, affective and behavioral components of BIDs between adolescent inpatients with AN or MDD recruited from two psychiatric hospitals and NCCs to investigate which body image components are specific to AN or MDD, and which represent normative adolescent development. The current study is part of a broader initiative focusing on AB in AN. Here, only the tasks and assessments relevant to the present hypotheses are discussed. Other tasks included a dot-probe task, an eyetracking AB task and a free-viewing task with eye-tracking; the results of these are reported elsewhere [19, 20, 32]. The study was approved by the ethical review board of the Ruhr-University Bochum (Registration-Number: 15-5541-BR) as well as the ethics committee of the Medical Faculty of the LMU Munich (Project-No. 814–16). The project was funded by the Swiss Anorexia Nervosa Foundation (project no. 54 − 15).

### Participants

A total of 108 female participants (aged 13–19 years) with a mean age of 15.62 years (*SD* = 1.45) were recruited. The AN group was recruited from two Child- and Youth- Psychiatric Hospitals in Germany (Hamm, Munich), while the MDD group and the NCC were recruited from a single site (Hamm). The NCC group was recruited from local schools via information events, flyers and posters in public places.

Clinical diagnoses for the inpatients were confirmed using a structured clinical interview (Kinder-DIPS; [33]; see below for details) according to DSM-5 criteria [34]. The AN group consisted of inpatients and outpatients with a primary diagnosis of AN. To be included in the MDD group, participants had to fulfill the criteria for a moderate or severe major depressive episode and must not have met the criteria for ED (currently or lifetime). Exclusion criteria for all groups were an intelligence quotient below 80, male sex, psychotic symptoms or disorders, bipolar disorders, substance abuse, pregnancy or neurological diseases, or insufficient knowledge of the German language. For the NCC, an additional exclusion criterion was any lifetime history of mental disorders. All participants and, if they were under 18, their guardians gave written informed consent in accordance with the Declaration of Helsinki.

To determine the required sample size, an a priori power analysis was performed (G*Power 3.1.9.2; [35]). With an assumed medium effect of *f* = 0.25, *α* = 0.05, and 1 − *β* = 0.95, a total of 66 participants (22 per group) was required. Our final sample comprised 92 adolescents (AN: *n* = 34, MDD: *n* = 28, NCC: *n* = 30); an additional 16 participants were excluded based on predefined exclusion criteria.

### Measures

#### Screening of cognitive processing speed

The German „Zahlen-Verbindungs-Test“ [36], a trail-making and speed test, was used as a screening of cognitive processing speed. The ZVT shows high to sufficient test reliability (*r* = .81 to 0.97; [37]) as well as moderate to high correlations (*r* = .40 to 0.83) with other intelligence tests [38, 39].

#### Diagnostic interview for mental disorders in children and adolescents (Kinder-DIPS)

To verify group specific inclusion and exclusion criteria regarding clinically relevant psychological diagnoses, the structured clinical interview Kinder-DIPS [33, 40] was administered to all participants. The Kinder-DIPS assesses the most prevalent mental disorders in children and adolescents according to DSM-5. It demonstrates high test-retest reliability (*κ* = 0.90-1.00; [41]), and interrater reliability [33], and is a well-established tool in German-speaking countries [42].

#### Stroop task

The body-related Stroop task (based on [28]) consisted of four different conditions, each presented on a different display. The stimuli included neutral words (e.g., “phase”, “print”), body-related words (e.g., “bony”, “broad”, “weight”), as well as positive (e.g., “dance”, “liberty”) and negative (e.g., “violence”, “poverty”) emotional words. The displays were presented on a computer in a randomized order, each containing 40 words printed in different colors (blue, red, yellow, green, and white). Participants were instructed to name the color of the words as quickly as possible while ignoring the semantic content. The dependent variables were the reaction times (RTs) per card and the number of errors (accuracy). RTs were measured manually using a stopwatch, starting from the onset of the first color named until the final word was completed.

#### Appraisal of their own body

After providing additional consent, photographs of the participants were taken in standardized underwear (front, side, and back views). For each view, participants were asked to select the three most attractive and the most unattractive body parts. Subsequently, they rated each of these nine body parts on a Visual Analogue Scale (VAS) ranging from 1 (very ugly) to 10 (very pretty). A mean score based on the three most attractive body parts from each view (from front, side, and back; nine ratings in total) was calculated and defined as “body appraisal”. Higher scores indicated a more positive body image, while lower scores reflected a more negative evaluation.

#### Beck-depression-inventory-2nd edition (BDI-II)

To assess the severity of depressive symptoms over the last two weeks, the German version of the internationally established self-report measure BDI-II [43] was used. It consists of 21 items with response categories ranging from 0 to 3. The resulting sum score allow for a differentiation between mild, moderate, and severe depression. The German version of the questionnaire has been validated in both adolescents and adult samples, demonstrating good internal consistency and validity [44, 45].

#### Eating disorder examination questionnaire (EDE-Q-II)

The German version of the EDE-Q-II [46, 47] was used to measure the severity of ED symptomatology. It consists of 22 items, each rated on a 7-point Likert-Scale (from 0 to 6). In addition to the total score, which indicates the general severity of ED symptoms over the past 28 days, the questionnaire comprises the four subscales “Restraint Eating”, “Eating Concern”, “Weight Concern” and “Shape Concern”. The German version shows high reliability (which was replicable in the current sample with a Cronbach’s *α* = 0.89) and retest reliability, as well as good convergent and discriminant validity [48].

#### Social appearance anxiety scale (SAAS)

The Social Appearance Anxiety Scale (SAAS; [49]; German version: [50]) is a 16-item self-report questionnaire employing a 5-point Likert scale ranging from 1 (not at all) to 5 (extremely). Participants are asked to rate their anxiety in social situations where their appearance might be evaluated. Higher scores indicate greater anxiety regarding appearance evaluation. The internal consistency in the present sample was excellent (Cronbach`s *α* = 0.96).

#### Body checking and avoidance questionnaire (BCAQ)

The Body Checking and Avoidance Questionnaire (BCAQ; [17]) consists of 27 items divided into three subscales: body checking, body avoidance and reassurance seeking. Items are rated on a 4-point Likert scale ranging from 1 (not at all true) to 4 (very true). Internal consistency in the present sample ranged from good to excellent (body checking: Cronbach`s *α* = 0.94, body avoidance: Cronbach`s *α* = 0.85, reassurance seeking: Cronbach`s *α* = 0.82).

### Study procedure

The participants attended three sessions. Initially, they were screened for IQ and psychiatric disorders, weight and height were measured, and standardized photographs of their bodies were taken. Furthermore, questionnaires were distributed to be completed at home. During the following two assessments, participants performed three behavioral tasks (a Stroop task, a dot probe task, and a free viewing task which included the appraisal of their own body) in a counterbalanced manner. In the original study, the participants underwent these tasks under two conditions: an anxiety mood induction and a low-anxiety control condition. Before, between and after the tasks, participants rated their current mood on VAS. A detailed description of this paradigm is provided by [19]. As the results of Radix et al. [19] demonstrated a comparable increase in anxiety, tension, and stress across both experimental conditions—suggesting the induction may not have been specific to anxiety—and found no impact of the anxiety induction on the AB in the dot-probe task, the present study does not distinguish between the two conditions. Consequently, only the low-anxiety control condition was analyzed. At the end of the experiment, participants watched a short clip from the movie “Happy feet” to induce a positive mood [51, 52]. Finally, the participants were debriefed and received 50€ as compensation.

### Statistical methods

All analyses were performed using SPSS version 29.0 for Windows. The two-sided level of significance was set at *α* = 0.05. First, RTs and accuracy data from the Stroop task were screened for potential outliers using boxplots. No participant met the criteria for exclusion (no extreme outliers defined as 2.5 interquartile ranges). Missing questionnaire data were imputed via Expectation Maximization (EM), provided that the data were missing completely at random (MCAR; *p* = 1.00) and only a few data points (≤ 4) were missing. One participant had to be excluded due to having more than four missing data points. Body mass percentiles were determined based on KIGGS data [53]. Interference scores for the Stroop task were calculated as a measure of AB by subtracting RTs of neutral words from RTs from body-related, positive and negative words, respectively. A positive interference score indicates an AB toward the respective word category. Finally, separate one-sample t-tests were performed to test significant differences from zero.

To examine differences between the three groups regarding demographic variables and descriptive statistics, univariate one-way analyses of variance (ANOVA) with Bonferroni-corrected post-hoc t- tests were conducted. One-way ANCOVAs were performed to test for group differences in BID attitudes. Age, BMI, and BDI scores were included as covariates to adjust for significant baseline imbalances between the groups. The dependent variables for these analyses included RT interference scores, Stroop task accuracy, body appraisal, shape concern, social appearance anxiety, body avoidance, body checking and reassurance seeking. Furthermore, bivariate correlations were performed to assess the associations between depressive symptoms (BDI-II sum score) and the three subscales of the EDE-Q-II (excluding the “Shape Concern” subscale) with body-related AB, body appraisal, shape concern, social appearance anxiety, body avoidance, body checking and reassurance seeking.

## Results

### Descriptive statistics

Descriptive statistics, as well as group differences regarding age, IQ, Body Mass Index (BMI), BMI percentiles (for participants under 18 years of age only), ED symptomatology, depressive symptoms, and the AB scores for positive and negative words, are presented in Table 1. The AN group was significantly younger than the NCC group. Furthermore, they exhibited higher IQ scores, a lower BMI, and lower BMI percentiles, as well as more pronounced ED symptomatology than both the MDD and NCC groups. The severity of depression (BDI-II) was highest in the MDD group; additionally, the AN group reported higher BDI scores group than the NCC. No significant difference was observed between the groups regarding AB for positive and negative words.

In the AN group, thirty-four participants presented with at least one comorbid diagnosis, including MDD (*n* = 13) and anxiety disorders (*n* = 5). In the MDD group, 28 participants fulfilled the criteria for at least one comorbid diagnosis, including anxiety disorders (*n* = 9), other emotional disorders of childhood (*n* = 4), mixed disorders of conduct and emotions (*n* = 4), gender dysphoria (formerly transsexualism; *n* = 1), and emotionally unstable personality disorder (impulsive type; *n* = 1).

**Table 1:** Descriptive statistics *M* (*SD*), one-way ANOVA and Bonferoni post-hoc tests

	AN (*n* = 34)	MDD (*n* = 28)	NCC (*n* = 30)	Teststatistics	Post-hoc tests
Age	15.03 (1.31)	15.82 (1.09)	16.10 (1.69)	*F*(2,89) = 5.16, *p* = .008, *η*^*2*^ = 0.10	AN = MDD AN ≠ NCC* MDD = NCC
IQ	109.91 (10.82)	99.45 (14.68)	102.4 (10.71)	*F*(2,89) = 6.37, *p* = 003, *η*^*2*^ = 0.13	AN ≠ MDD* AN ≠ NCC* MDD = NCC
BMI	16.62 (1.60)	23.95 (5.26)	22.12 (3.93)	*F*(2,88) = 31.23, *p* < .001, *η*^*2*^ = 0.42	AN ≠ MDD** AN ≠ NCC** MDD = NCC
Body mass index percentiles***	7.82 (10.16)	60,89 (30.65)	48.88 (29.58)	*F*(2, 81) = 39.68, *p* < .001, *η*^*2*^ = 0.50	AN ≠ MDD** AN ≠ NCC** MDD = NCC
SUM_EDE-Q	3.05 (1.80)	1.89 (1.62)	1.09 (1.04)	*F*(2,89) = 13.10, *p* < .001, η^2^ = 0.22	AN ≠ MDD* AN ≠ NCC** MDD = NCC
SUM_BDI	23.79 (15.22)	31.90 (11.65)	6.17 (5.67)	*F*(2,89) = 36.95, *p* < .001, *η*^*2*^ = 0.45	AN ≠ MDD* AN ≠ NCC** MDD ≠ NCC**
AB_negative words	0.67 (4.79)	0.59 (4.35)	2.06 (4.62)	*F*(2,87) =0.96, *p* = .39, η^2^ = 0.02	
AB_positive words	0.89 (4.30)	− 0.42 (4.55)	0.48 (3.36)	*F*(2,87) =0.96, *p* = .47, η^2^ = 0.02	

AN = Patients with Anorexia Nervosa, MDD = Patients with Depression, NCC = Non-Clinical Control Group, IQ = Intelligence Quotient, BMI = Body Mass Index, EDE-Q = Eating Disorder Examination Questionnaire-2nd, BDI-II = Beck Depression Inventar-2nd edition, AB = Attentional Bias, * *p* < .05; ** *p* < .001***; based on the KIGGS-data [53]

### Group differences in body image attitudes

**Fig. 1:**
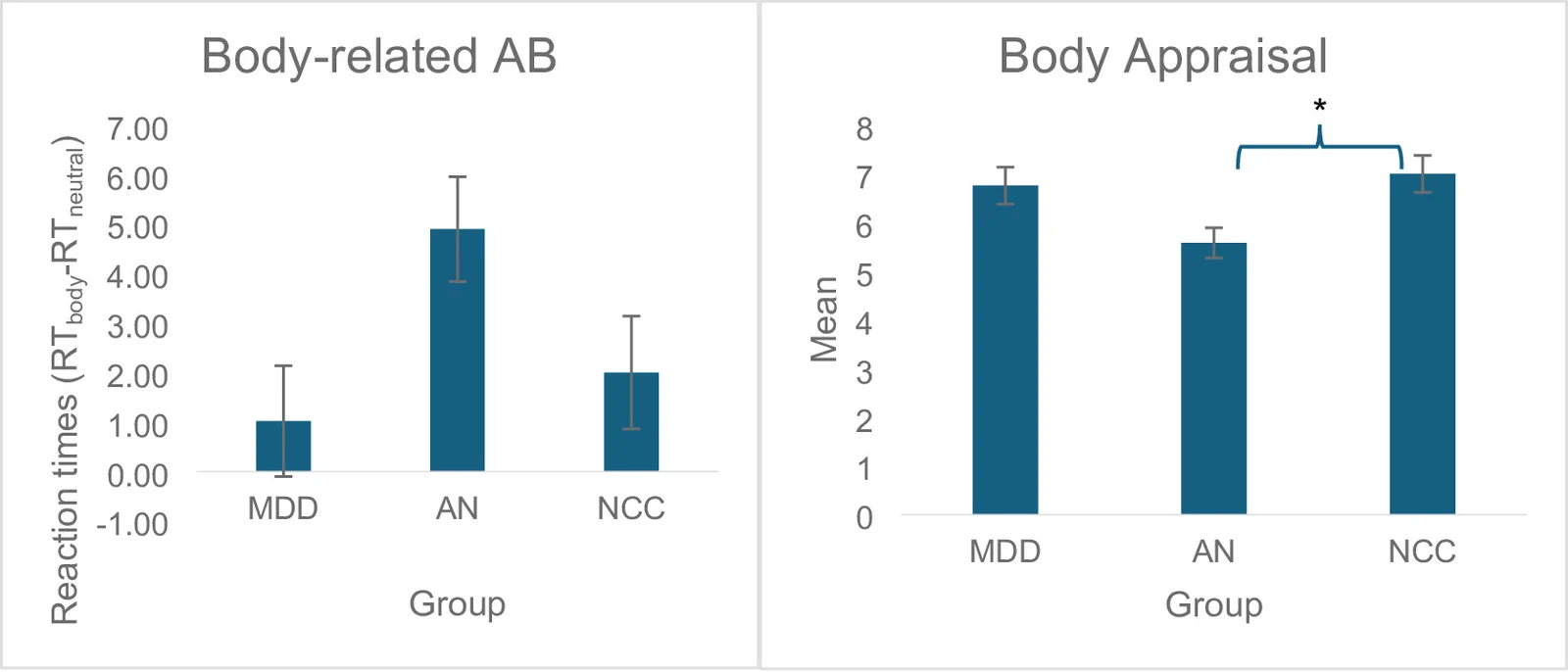
Reaction Times of the Body-Related AB and Mean of Body Appraisal Across MDD, AN, and NCC groups. Error bars represent standard errors (SE). MDD = Major Depressive Disorder, AN = Anorexia Nervosa and NCC = Non-clinical controls. *p < .05

Group differences are displayed in Table 2. The three groups did not differ significantly regarding body-related AB. However, separate one-sample *t*-tests indicated that AB scores were significantly greater than zero in both the AN group, *t*(32) = 4.52, *p* < .001, *d* = 6.07, and the NCC group, *t*(29) = 2.31, *p* = .03, *d* = 3.64. In contrast, the MDD group showed no significant body-related AB, *t*(26) = 1.99, *p* = .06. Regarding body appraisal, no significant difference was found between the two clinical groups, whereas the AN group differed significantly from the NCC group (see Fig. 1 for reaction times and mean appraisal scores). Furthermore, the AN group reported significantly higher levels of shape concerns, social appearance anxiety, body avoidance, and body checking compared to the MDD group. Notably, no significant differences emerged between the NCC and the clinical groups on these measures. In the ANCOVAs, BMI and BDI scores served as significant covariates for shape concerns, *F*(1,85) = 8.66, *p* = .01, *η*^*2*^ = 0.09; *F*(1,85) = 19.33, *p* < .001, *η*^*2*^ = 0.19, social appearance anxiety, *F*(1,85) = 17.31, *p* < .001, *η*^*2*^ = 0.17; *F*(1,85) = 33.27, *p* < .001, *η*^*2*^ = 0.28, and body avoidance, *F*(1,85) = 17.53, *p* < .001, *η*^*2*^ = 0.17; *F*(1,85) = 31.18, *p* < .001, *η*^*2*^ = 0.27. Additionally, body appraisal was significantly correlated with BMI, *F*(1,65) = 5.05, *p* = .03, while body checking was significantly associated with both BDI scores and age, *F*(1,85) = 11.66, *p* < .001, *η*^*2*^ = 0.12; *F*(1,85) = 4.46, *p* = .04, *η*^*2*^ = 0.05. The three groups did not differ regarding AB accuracy, *F*(2,83) = 0.72, *p* = .49, nor did they differ with regard to reassurance seeking.

**Table 2:** Comparisons between the three groups concerning the various components of body image were conducted using adjusted means (M) and standard errors (SE), controlling for age, body mass index and depressive symptoms

	AN (*n* = 33)	MDD (*n* = 28)	NCC (*n* = 30)	Teststatistics	Post-hoc tests
Body-related AB	4.90 (1.06)	1.02 (1.12)	2.00 (1.14)	*F*(2,83) = 3.02, *p* = .054, *η*^*2*^ = 0.07	AN = MDD AN = NCC MDD = NCC
Body appraisal	5.58 (0.31)	6.76 (0.38)	7.00 (0.38)	*F*(2,65) = 4.08, *p* = .022, *η*^*2*^ = 0.11	AN = MDD AN ≠ NCC* MDD = NCC
EDE-Q_Shape concern	5.04 (0.34)	2.75 (0.36)	3.38 (0.37)	*F*(2,85) = 9.82, *p* < .001, *η*^*2*^ = 0.19	AN ≠ MDD** AN ≠ NCC* MDD = NCC
SAAS	49.83 (2.83)	36.46 (3.01)	41.66 (3.09)	*F*(2, 85) = 4.73, *p* = .010, *η*^*2*^ = 0.10	AN ≠ MDD AN = NCC MDD = NCC
BCAQ_avoidance	25.26 (1.26)	20.34 (1.34)	21.22 (1.38)	*F*(2, 85) = 3.54, *p* = .033, *η*^*2*^ = 0.10	AN ≠ MDD* AN = NCC MDD = NCC
BCAQ_checking	30.24 (1.89)	18.58 (2.01)	23.49 (2.06)	*F*(2,85) = 8.01, *p* < .001, *η*^*2*^ = 0.16	AN ≠ MDD** AN = NCC MDD = NCC
BCAQ_reassuring	4.65 (2.56)	4.95 (2.50)	4.54 (1.96)	*F*(2,85) = 1.77, *p* = .18, *η*^*2*^ = 0.04	

These were analyzed via one-way ANCOVAs with Bonferroni-corrected post-hoc tests

AN = Patients with Anorexia Nervosa, MDD = Patients with Depression, NCC = Non-Clinical Controls, AB = Attentional Bias, EDE-Q = Eating Disorder Examination Questionnaire-2nd, SAAS = Social Appearance Anxiety Scale, BCAQ = Body Checking and Avoidance Questionnaire, * *p* < .05; ** *p* < .001

### Associations between eating-disorder symptomatology, depression, and body image components

Bivariate correlations between depressive symptoms (BDI-II sum score) and the ED symptomatology (three subscales of the EDE-Q-II, excluding shape concern) with body-related AB, body appraisal, shape concern, social appearance anxiety, body avoidance, body checking, and reassurance seeking are presented in Table 3.

**Table 3:** Bivariate correlations between eating-disorder and depressive symptoms with the body-related AB, body appraisal, shape concerns, social appearance anxiety, avoidance, checking and reassurance

	SUM_EDE-Q _(without the subcale shape concerns)_	SUM_BDI-II
SUM_BDI-II	0.54**	1
Body-related AB	0.19	0.10
Body appraisal	− 0.44**	− 0.30*
Shape concern	0.88**	0.53**
SUM_SAAS	0.60**	0.63**
BCAQ_avoidance	0.69**	0.64**
BCAQ_checking	0.80**	0.38**
BCAQ_reassurance	0.51**	0.30**

BDI-II = Beck Depression Inventar-2nd edition, EDE-Q-II = Eating Disorder Examination Questionnaire-2nd edition, AB = Attentional Bias, SAAS = Social Appearance Anxiety Scale, BCAQ = Body Checking and Avoidance Questionnaire, * *p* < .05, ** *p* < .001

## Discussion

The present study compared cognitive, affective and behavioral components of BIDs between adolescent inpatients with AN or MDD and NCCs to investigate which body image components are specific to AN or MDD, and which represent normative adolescent development. Results indicated that the AN group and the NCC revealed a significant body-related AB, whereas the MDD group did not. In terms of body appraisal, the clinical groups rated their bodies more negatively than the NCC. On shape concerns, anxiety, and body avoidance and checking, the AN group showed elevated scores that distinguished them significantly from the MDD group, whereas the NCC only differed significantly from the AN group in terms of shape concerns. Consequently, while these clinical groups share similarities in their negative subjective body ratings, their automatic cognitive processing and behavioral manifestations appear to diverge.

With regard to the body-related AB (based on reaction times), contrary to our hypothesis and to previous findings [19, 20], adolescent inpatients with MDD showed no significant AB to body-related words, whereas both the AN and NCC groups revealed a significant AB. One explanation for the different results could be the different stimuli used in these two tasks. While pictorial stimuli are directly processed and closely linked to amygdala activation [54], semantic stimuli must be processed indirectly via the semantic system [55]. Thus, the processing of pictorial stimuli is faster and may be sufficiently salient and threatening to capture the attention of adolescents with MDD.

In contrast, the semantic processing of body-related words does not appear to significantly challenge their self-concept. This discrepancy suggests that BID in MDD may not be as deeply ingrained as it is in patients with AN. Consequently, BIDs in MDD might be more transient or stimulus-dependent, rather than reflecting a core, stable manifestation of the disorder. Interestingly, the NCC group also showed a significant body-related AB, albeit descriptively less pronounced than in the AN group. This could represent the normative developmental salience of body-related stimuli in adolescence, a finding consistent with previous research highlighting heightened appearance focus and increased social media exposure in this age group [2].

Regarding body appraisal, the analyses showed equally negative body appraisals in the MDD and AN group, whereas the NCC significantly rated their bodies more positively than the AN group. These findings indicate that negative body appraisal is a transdiagnostic issue that potentially contributes to body dissatisfaction. Given that previous studies have consistently found strong positive associations between body dissatisfaction and depressive symptoms—identifying body dissatisfaction as a risk factor for MDD (e.g., [23])—these results align with existing research. Since body dissatisfaction increases vulnerability to MDD, prevention and treatment programs should include the promotion of body satisfaction and body acceptance in youth with MDD.

With respect to shape concerns, appearance-related anxiety, body checking, body avoidance and checking, the MDD group exhibited significant differences compared to the AN group across most subjective BID components, even when controlling for BDI scores and BMI. Since depressive symptoms served as a significant covariate for nearly all subjective measures, BIDs in the MDD group may be interpreted as manifestations of the cognitive triad [26]. In this framework, the body is not necessary the primary source of pathology, as is typical for AN, but rather might be a domain which reflects the negative self-schema.

Regarding the subjective BID components, the NCC group differed significantly from the AN group in terms of shape concerns, yet showed no significant differences in appearance-related anxiety, body checking, or body avoidance. Descriptively, the NCC group’s scores were situated between those of the AN and MDD groups. This lack of significant differences in anxiety and behavioral manifestations suggests that these components may be particularly sensitive to the normative developmental challenges of adolescence.

As adolescence is characterized by rapid physical and hormonal changes alongside high pressure to conform to societal beauty ideals [2], a certain level of body-related anxiety, avoidance, and checking behavior may be widespread even among healthy adolescents. This potentially blurs the line between normative and clinical populations in standardized self-report measures. However, since the NCCs revealed significantly lower shape concerns and a more positive body appraisal than the AN group, it can be argued that while they may engage in similar body-related behaviors, the cognitive-emotional weight and pathological significance they attach to their body is substantially lower. For the NCC group, these behaviors may reflect a temporary developmental preoccupation, whereas for the AN group, they represent a deeply entrenched and distressing core symptom of the disorder. In summary, the three groups exhibited distinct profiles across the various BID components. While pathological body appraisal (negative ratings) appears to be a transdiagnostic feature shared by both clinical groups, conspicuous scores on affective and behavioral components remain specific to AN. In the MDD group, these mechanisms may be less ‘hard-wired’; instead, they might represent state-dependent manifestations of a negative self-schema that may alleviate during remission as overall depressive symptoms diminish. Given the cross-sectional nature of the present study, this hypothesis remains tentative and should be addressed in future longitudinal research.

Furthermore, while all subjective BID components correlated moderately to highly with both eating disorder psychopathology and depressive symptoms, the body-related AB stood out as the only component not significantly associated with these measures. This lack of correlation suggests that automatic cognitive processing (AB) may capture a distinct facet of body image that operates independently of conscious, self-reported psychopathology.

### Strengths and limitations

While previous studies often explored only a single BID component, the present study integrates multiple components to compare BIDs across adolescents with AN, MDD, and NCCs. A major strength is the inclusion of two clinical samples. Although comorbidities were present (comorbid depression in the AN group and comorbid anxiety in both clinical groups), statistical results confirmed that depressive symptoms were significantly higher in the MDD group, justifying the inclusion of this specific clinical sample.

The Stroop task was employed to examine whether previous findings generalize from purely perceptual reactions to semantic processing. This allowed us to investigate whether the AB is deeply rooted in abstract knowledge structures and the self-concept, rather than being merely a reaction to visual stimuli. However, body-related AB in the Stroop task did not significantly associate with disorder symptoms, suggesting it may not be a sufficiently valid measure of BID in this context. A limitation of this study is the relatively small size of the clinical subgroups. While sufficient for primary group comparisons (ANOVAs), these sample sizes may have constrained the detection of subtle effects in bivariate correlations. Furthermore, the inclusion of additional covariates may have reduced the statistical power by consuming degrees of freedom, which could account for the non-significant differences observed between the AN and NCC groups. Larger clinical samples, especially in the MDD group, would be beneficial for future research to increase the robustness and reliability of transdiagnostic comparisons. Finally, as this study is cross-sectional, the results should be interpreted with caution and have to be explored in further longitudinal studies.

## Conclusions

The finding that healthy adolescents revealed no significant differences in body-related behaviors compared to the AN group suggests that prevention programs targeting checking and avoidance behavior may reduce the risk of developing disordered eating behaviors. Implemented early, such programs could mitigate body dissatisfaction and prevent the progression into clinical eating disorders. Since subjective dissatisfaction appears to be elevated in both AN and MDD, transdiagnostic prevention approaches are justified. A meta-analysis investigating the secondary effects of body dissatisfaction interventions on depressive symptoms found that these interventions yielded effects comparable to those specifically targeting depression [56]. Consequently, as body dissatisfaction interventions may mitigate or prevent adolescent depressive symptoms, future research should prioritize the development and evaluation of these targeted approaches.

## Data Availability

The datasets and materials used and/or analysed during the current study are available from the corresponding author on reasonable request.

## Abbreviations

AB: Attentional bias
AN: Anorexia nervosa
BID-II: Beck-Depression-Inventory-2nd edition
BID: Body image disturbance
BCAQ: Body checking and avoidance questionnaire
BMI: Body Mass Index
ED: Eating disorder
EDE-Q-II Eating: Disorder examination questionnaire-2nd edition
EM: Expectation maximization
Kinder-DIPS: Diagnostic interview for mental disorders in children and adolescents
MDD: Major depressive disorder
NCC: Non-clinical controls
RT: Reaction time
SAAS: Social appearance anxiety scale
VAS: Visual analogue scale
ZVT: Zahlen-Verbindungs-Test
